# “Auto-anti-IgE”: Naturally occurring IgG anti-IgE antibodies may inhibit allergen-induced basophil activation

**DOI:** 10.1016/j.jaci.2014.06.029

**Published:** 2014-12

**Authors:** Yih-Chih Chan, Faruk Ramadani, Alexandra F. Santos, Prathap Pillai, Line Ohm-Laursen, Clare E. Harper, Cailong Fang, Tihomir S. Dodev, Shih-Ying Wu, Sun Ying, Christopher J. Corrigan, Hannah J. Gould

**Affiliations:** a Department of Asthma, Allergy and Respiratory Science and Randall Division of Cell and Molecular Biophysics, King's College London; the Medical Research Council and Asthma UK Centre, Allergic Mechanisms in Asthma, London; the Department of Paediatric Allergy, Guy's and St Thomas' National Health Service Foundation Trust, London, United Kingdom; b Immunoallergology Department, Coimbra University Hospital, Coimbra, Portugal

**Keywords:** Asthma, autoantibodies, IgE, basophil activation, basophil inhibition, AA, Atopic asthmatic subjects, ANA, Anti-nuclear autoantibodies, APC, Allophycocyanin, BAT, Basophil activation test, FACS, Fluorescence-activated cell sorting, FITC, Fluorescein, HDM, House dust mite, HRP, Horseradish peroxidase, MFI, Mean fluorescence intensity, MP, Milk powder, NAA, Non-atopic asthmatic subjects, NAC, Non-atopic controls, PBS-T, Phosphate-buffered saline/Tween 20, PE, R-Phycoerythrin, PEFR, Peak expiratory flow rate, RT, Room temperature, SPT, Skin prick test

## Abstract

**Background:**

Naturally occurring IgE-specific IgG autoantibodies have been identified in patients with asthma and other diseases, but their spectrum of functions is poorly understood.

**Objective:**

Address the hypothesis that: (i) IgG anti-IgE autoantibodies are detectable in the serum of all subjects but elevated in asthmatic patients regardless of atopic status as compared with controls; (ii) some activate IgE-sensitized basophils; and (iii) some inhibit allergen-induced basophil activation.

**Methods:**

IgE-specific IgG autoantibodies were detected and quantified in sera using ELISA. Sera were examined for their ability to activate IgE-sensitized human blood basophils in the presence and absence of allergen using a basophil activation test, and to inhibit allergen binding to specific IgE on a rat basophilic cell line stably expressing human FcεRI.

**Results:**

IgG autoantibodies binding to both free and FcεRI-bound IgE were detected in patients with atopic and non-atopic asthma, as well as controls. While some were able to activate IgE-sensitised basophils, others inhibited allergen-induced basophil activation, at least partly by inhibiting binding of IgE to specific allergen.

**Conclusion:**

Naturally occurring IgG anti-IgE autoantibodies may inhibit, as well as induce, basophil activation. They act in a manner distinct from therapeutic IgG anti-IgE antibodies such as omalizumab. They may at least partly explain why atopic subjects who make allergen-specific IgE never develop clinical symptoms, and why omalizumab therapy is of variable clinical benefit in severe atopic asthma.

IgE is thought to participate in host defense, but it also has a central role in the pathogenesis of allergy and asthma.^1^ Basophils and mast cells express the IgE high affinity receptor FcεRI and mediate type I hypersensitivity reactions^2^ following cross-linking of surface IgE-FcεRI complexes by multivalent antigens, including allergens, causing activation/degranulation^3^ and clinical symptoms.

Previous studies in humans have identified the production of autoantibodies of the IgG or IgM class that bind specifically to IgE or FcεRI. Some are able to activate basophils and mast cells independently of antigens.^4^ Autologous serum skin tests,^5^ measurement of histamine release from blood basophils,^6^ and, more recently, basophil activation assays using flow cytometry^7^ have been used to detect potential proinflammatory activities of these autoantibodies. Most IgE-specific IgG autoantibodies are of the IgG_1_ or IgG_4_ isotype^8^ and appear to recognise 2 epitopes within the IgE Cε2 and Cε4 domains.^9^ FcεRI-specific IgG autoantibodies of the IgG_1_ and IgG_3_ isotypes have been described predominantly in patients with chronic urticaria, while IgG_2_ and IgG_4_ isotypes have been described in other autoimmune disorders.^10^ Autoantibodies against IgE or FcεRI have been detected in various diseases, including atopic dermatitis,^11^, ^12^ asthma,^13^ and autoimmune disorders.^10^, ^14^

Two general, striking features of these studies stand out. First, not all of these autoantibodies show proinflammatory activity,^15^, ^16^ at least as detected by the aforementioned assays, so their activities do not reflect their concentrations,^17^ with 1 study hinting at a possible regulatory role.^8^ Secondly, IgE-specific and FcεRI-specific autoantibodies are also detectable in apparently healthy individuals.^18^, ^19^

Consequently, we set out to examine the possibility that IgG anti-IgE autoantibodies may in some individuals exert an anti-inflammatory, rather than a proinflammatory, effect. We elected to focus on asthma as an archetypal disease involving IgE-mediated mechanisms, in which exogenous IgG anti-IgE (omalizumab) has a proven therapeutic role at least in some individuals, and on anti-IgE rather than anti-FcεRI autoantibodies, for the same reason. We hypothesized that: (i) IgG anti-IgE autoantibodies are detectable in the serum of all subjects but elevated in asthmatic subjects regardless of atopic status as compared with controls; (ii) some of these antibodies can activate IgE-sensitized basophils; (iii) some of these antibodies do not activate IgE-sensitized basophils and can, furthermore, inhibit allergen-induced activation.

To address these hypotheses, we developed and calibrated an *in vitro* assay to detect and quantify IgG anti-IgE autoantibodies in the serum of asthmatic subjects and controls. We then tested the ability of these sera to activate or inhibit IgE-sensitized blood basophils from a single atopic donor in the presence and absence of allergen and finally utilized a rat basophilic cell line stably expressing human FcεRI bound to in-house manufactured monoclonal IgE directed against the *Phl p 7* component of timothy grass allergen^20^ to examine the ability of sera to inhibit the binding of these cells to specific allergen.

## Methods

### Participants

Atopic asthmatic (AA), non-atopic asthmatic (NAA), and non-atopic non-asthmatic control (NAC) subjects were recruited from the departmental asthma clinic at Guy's Hospital, London and databases or through advertisements. All participants gave written informed consent to participate in the study, which was approved by a local research ethics committee. A diagnosis of asthma was accepted based on relevant symptoms and 1 of the following criteria: i) documented ≥12% reversibility of FEV_1_ or PEFR in response to inhaled bronchodilators (nebulized salbutamol 2.5 mg and ipratropium 500 μg); ii) documented ≥8% variability of PEFR during a 24-hour period or ≥20% variability over a period of 1 to 2 weeks; or iii) a positive mannitol bronchial challenge test (Osmohale; Pharmaxis Pharmaceuticals Ltd, Burnham, United Kingdom). Non-asthma was defined as absence of relevant symptoms, with FEV_1_ in the normal range. Atopy was defined as a positive *in vitro* IgE test to 1 or more of the following local aeroallergens: mixed grass, mixed tree, mixed mould, house dust mite (HDM), and cat and dog dander. Non-atopy was defined as negative *in vitro* IgE tests (Phadia ImmunoCAP; Thermo Fisher Scientific, Uppsala, Sweden) (Grade 0 or ≤0.35 kU/L) to the same aeroallergens. The median and range % predicted FEV_1_ of the AA and NAA subjects and the NAC at the time of the study were 97 (64-143), 73 (40-119) and 106 (94-128), respectively. All patient sera were screened for anti-nuclear autoantibodies (ANA) using commercially available ANA-ELISA kits (Abnova, Taipei City, Taiwan) according to the manufacturer's instructions.

### ELISA

Maxisorp plates (Thermo Fisher Scientific) were coated either with 0.5 μg/mL of recombinant IgE (Abcam, Cambridge, United Kingdom) to measure IgG anti-IgE or with anti-human IgG (diluted 1:1000; AbD Serotec, Oxford, United Kingdom) to measure total IgG, in 50 mM carbonate buffer pH 9.6 at 4°C overnight. Non-specific binding was blocked with SuperBlock blocking buffer (Thermo Fisher Scientific) or 3% milk powder (MP) in PBS-T at room temperature (RT) for 2 hours, and the plates then washed 3 times with PBS-T. Test sera were added at 1:5 to 1:20 dilutions in 1% MP-PBS-T and the plates incubated at RT for 1 hour then washed 3 times with PBS-T and incubated with anti-human IgG-HRP (diluted 1:10000 in 1% MP-PBS-T; Sigma-Aldrich, St Louis, Mo) for 1 hour at RT. The plates were washed again 3 times and the color reaction developed using TMB solution (R&D Systems, Minneapolis, Minn). The reaction was stopped by addition of 1.8 M H_2_SO_4_ and absorbance read at 450 nm using a Multiskan EX plate reader (Thermo Fisher Scientific). The assay was calibrated using a commercial IgG anti-IgE monoclonal antibody (omalizumab; Novartis, Surrey, United Kingdom) or human IgG (Sigma-Aldrich). All samples were measured at least in duplicate. To further allow for any non-specific IgG binding to the plates and determine the threshold of sensitivity of the anti-IgE ELISA (0.27 ng/mL), commercial human IgG (Sigma-Aldrich) was used at a relatively high concentration (2 μg/mL) in place of sera as a negative control and the threshold defined as 3 standard deviations above the mean resulting absorbance.

The binding of IgG anti-IgE autoantibodies to FcεRI-bound IgE was determined by pre-coating the ELISA plate with recombinant FcεRIα (0.5 μg/mL; R&D Systems) in carbonate buffer at 4°C overnight. After washing 3 times with PBS-T and blocking with 3% MP in PBS-T for 2 hours at RT, the plates were incubated overnight at 4°C with recombinant IgE (0.5 μg/mL in 1% MP in PBS-T). Plates were washed 3 times with PBS-T before the addition of the test sera and the assay developed as described above. Each sample was assayed at least in duplicate.

### Basophil activation test (BAT) by flow cytometry

PBMC from a single atopic non-asthmatic donor with total serum IgE >150 IU/mL and sensitized to HDM as determined by skin prick testing were isolated using Ficoll-Paque PLUS (GE Healthcare, Amersham, United Kingdom) and re-suspended in HBSS (Sigma-Aldrich) to a final concentration of 2 x 10^7^ cells/mL.

To test the activity of subject sera in the BAT assay, 1 × 10^6^ PBMC (50 μL of suspension) were incubated with sera diluted 1:2 in BAT buffer to 50 μL (HBSS with 2 mM CaCl_2_) for 30 minutes at 37°C and the reaction stopped by adding 2 mL of FACS buffer containing 2 mM EDTA on ice. The donor's own serum was used as a baseline control. As a positive control, instead of sera, PBMC were activated using polyclonal anti-IgE (Dako, Glostrup, Denmark) in the concentration range 6 ng/mL to 6 μg/mL in BAT buffer.

Flow cytometric analysis of basophil activation was adapted from a previously published protocol.^21^ PBMC were stained with anti-CD203c-phycoerythrin (Clone NP4D6; BioLegend, San Diego, Calif) and anti-CD63-allophycocyanin (Clone MEM-259; BioLegend) using the manufacturer's recommended concentrations in FACS buffer (PBS containing 2% FBS) for 30 minutes on ice, washed in FACS buffer and analyzed using a BD FACSCalibur (BD Biosciences, San Diego, Calif). Basophil activation was defined as the percentage of CD203c^+^ basophils expressing the activation marker CD63 compared with the baseline control.

### Allergen-induced basophil activation

To determine the effect of subjects' sera on allergen-induced basophil activation, we pre-incubated 10^6^ PBMCs in 50 μL of BAT buffer from a single atopic donor sensitized to HDM with the sera (diluted 1:2 in BAT buffer to 50 μL), purified anti-IgE, or the donor's own serum as a baseline control for 30 minutes at 37°C. The cells were then washed and resuspended in 50 μL BAT buffer prior to the addition of serial dilutions of the major HDM antigen *Der p 2* from 300 to 3 ng/mL for 30 minutes at 37°C. The reactions were stopped by adding 2 mL of FACS buffer containing 2 mM EDTA on ice, and basophil activation was determined as described above.

### Depletion of total IgG and IgE binding proteins from sera

To deplete total IgG from sera, 500 μL samples were incubated with an equal volume of protein G Sepharose (Sigma-Aldrich) at 4°C overnight in a mini Bio-Spin chromatography column (Bio-Rad, Hercules, Calif). The flow-through was recovered and re-incubated with fresh protein G Sepharose at 4°C overnight. IgG was measured in the final recovered flow-through by ELISA.

**Fig E1:**
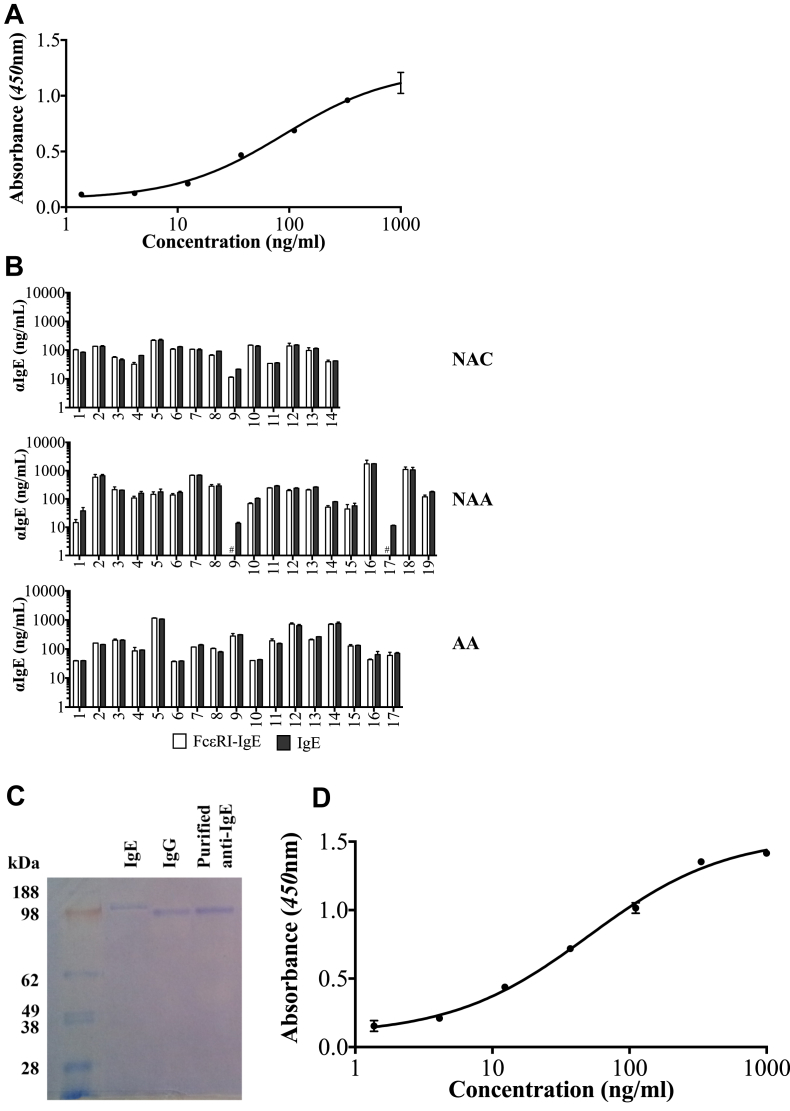
Representative standard concentration curve for the IgG anti-IgE ELISA using omalizumab **(A)**. Comparison of the capacity of IgE-specific IgG autoantibodies to bind to “free” IgE *(dark bars)* and FcεRI-bound IgE *(white bars)* # analyte below detection limit of assay. Bars show mean/SD of 3 experiments using duplicate samples **(B)**. SDS-PAGE showing the purified IgG anti-IgE from serum compared with recombinant IgE and IgG **(C)**. Representative standard concentration curve for the IgG anti-FcεRI-bound ELISA using anti-IgE purified from human sera **(D)**.

To deplete IgE-binding proteins, IgE was cross-linked to cyanogen bromide (CNBr)–activated Sepharose 4B (Sigma-Aldrich) according to the manufacturer's instructions. Briefly, 100 μg of recombinant IgE anti-NP-BSA (manufactured in-house) were mixed with 100 μL of 1 mM HCl-swollen CNBr-activated Sepharose in coupling buffer (0.1 M NaHCO_3_ buffer containing 0.5 M NaCl) at 4°C overnight in a mini Bio-Spin chromatography column, then washed with coupling buffer. Unconjugated sites were blocked with 0.2 M glycine buffer pH 8.0 for 2 hours at RT. After extensive washing with 5 cycles of the coupling buffer and 0.1 M acetate buffer pH 4, the IgE-coupled Sepharose was ready to be used. IgE-coupled Sepharose (50 μL) was mixed with subjects' sera (3 mL) at 4°C overnight. The eluate containing non-IgE binding antibodies was stored at −20°C for later analysis. Proteins bound to the IgE-coupled Sepharose were then eluted with 100 μL of 0.2 M glycine pH 2.5 into an equal volume of 100 mM Tris buffer pH 8.0. The eluted solutions were dialyzed in PBS at 4°C overnight, then mixed with protein G Sepharose (1:1) at 4°C overnight to capture IgG antibodies. The IgG anti-IgE antibodies were eluted off the protein G Sepharose with glycine, dialyzed with PBS as above, then quantified by ELISA and run on 10% SDS-PAGE under non-reducing conditions compared with recombinant IgE and IgG (Sigma-Aldrich). No contaminating IgE was found in purified antibodies, as determined by total IgE ELISA (individual data not shown; see Fig E1, *C*, in this article's Online Repository at www.jacionline.org).

### Effects of sera on allergen binding to IgE-bound basophils

To determine the effects of our test sera on allergen binding to IgE on the surface of basophils, we used pure, recombinant *Phl p 7*–specific IgE subcloned from an existing IgG_4_ clone isolated in-house^20^ by replacement of the Cγ4 constant region with that of IgE (Cε; Dodev et al; manuscript in press). Cells of the RBL-SX38 rat basophilic cell line, which stably express human FcεRI,^22^ were cultured in RPMI-1640 medium (Sigma-Aldrich) containing 10% FBS (Sigma-Aldrich), 1% Penicillin-Streptomycin-Glutamine (Life Technologies, Carlsbad, Calif) and 1 mg/mL Genticin (Life Technologies). RBL-SX38 cells were harvested and resuspended in BAT buffer at 2 × 10^7^ cells/mL, then incubated with *Phl p 7*–specific IgE at a concentration of 0.5 μg/mL in 50 μL BAT buffer for 30 minutes at 37°C. Aliquots of 10^6^ cells were washed with HBSS and incubated with 50 μL aliquots of the sera (diluted 1:2 in BAT buffer) for 30 minutes at 37°C. After further washing with HBSS, the cells were resuspended and incubated with 50 μL of 1 μg/mL of in-house produced, freshly biotinylated *Phl p 7* diluted in BAT buffer for 30 minutes at 37°C. Surface-bound IgE was detected using anti-IgE-FITC (Vector Laboratories, Burlingame, Calif) and *Phl p 7* binding to this IgE using streptavidin-APC (BioLegend), with analysis by flow cytometry.

### Statistical analysis

Statistical analysis was performed using 2-way ANOVA with Bonferroni correction or as otherwise stated. A *P* value of < .05 was considered significant (*P < .05, **P < .01, ***P < .001).

## Results

### Identification of naturally occurring IgG class autoantibodies against free and FcεRI-bound IgE

**Fig 1:**
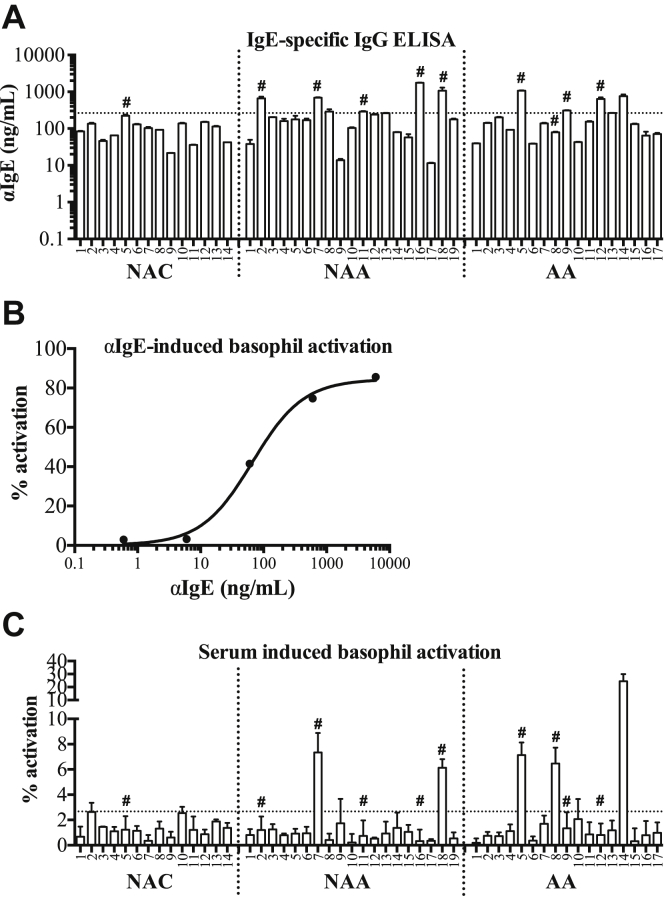
Concentrations of IgG anti-IgE autoantibodies in sera from non-atopic controls, non-atopic asthmatic subjects, and atopic asthmatic subjects **(A)**. *Dotted line* shows 95% confidence limit of the range in controls. Response of blood basophils to polyclonal anti-IgE *in vitro***(B)**. Basophil-activating activity of sera **(C)**. *Dotted line* shows detection threshold. Samples marked *#* were used for later experiments. *Bars* represent the mean/SD of at least 3 independent experiments.

Serum samples were screened for IgG-class antibodies specific for IgE using an in-house, custom ELISA as described in the Methods section. IgG anti-IgE autoantibodies were detected above the threshold of sensitivity of the assay after allowing for non-specific binding in all subjects in all 3 groups, with concentrations ranging from 22 to 223 ng/mL, 11 to 1761 ng/mL, and 39 to 1070 ng/mL in the NAC, NAA, and AA, respectively (Fig 1, *A*).

Omalizumab was used as a standard to calibrate the ELISA (see Fig E1, *A*). A “normal” range for these measurements has yet to be defined, but some serum samples from the asthmatic subjects clearly showed concentrations of IgG anti-IgE in excess of the 95% confidence limit of the measurements in the normal controls (Fig 1, *A*). Nevertheless, many did not, and overall, there was no significant variation in the concentrations of these antibodies between the 3 groups. There were no systematic differences in binding of any of the IgE-specific autoantibodies to IgE alone and FcεRI-bound IgE, although in 2 of the samples from the non-atopic asthmatic subjects (NAA9 and NAA17), binding of the autoantibodies to FcεRI-bound IgE was below the lower limit of detection of the assay and therefore technically unquantifiable (see Fig E1, *B*). Purified IgG anti-IgE autoantibody from a single patient's serum was used as a standard to calibrate the ELISA for measuring FcεRI-bound IgE, because omalizumab does not bind to FcεRI-bound IgE. Gel electrophoresis confirmed that this antibody, as well as antibodies from the remainder of the study subjects, was pure and, in particular, free of contaminating IgE (see Fig E1, *C* and *D*).

**Fig E2:**
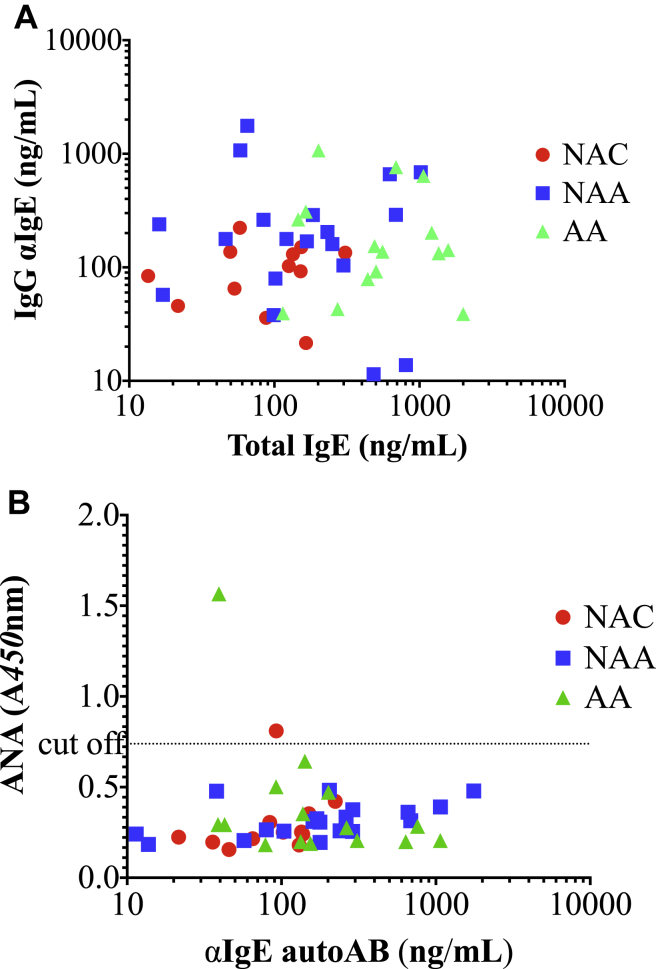
Comparison of serum total IgE and IgG anti-IgE autoantibody concentrations in all study subjects **(A)**. Spearman *r* = 0.08578; *P* > .5. Comparison of serum concentrations of IgG anti-IgE antibodies with ANA in all study subjects **(B)**. *Dotted line* shows the threshold for a positive ANA result. Spearman *r* = −0.02621; *P* > .5.

Concentrations of IgG anti-IgE antibodies were compared with those of total IgE and anti-nuclear autoantibodies in all of the study subjects (Fig E2, *A* and *B*, in this article's Online Repository at www.jacionline.org). No correlation was observed in either case.

### Basophil activation by sera containing IgG anti-IgE autoantibodies

**Fig E3:**
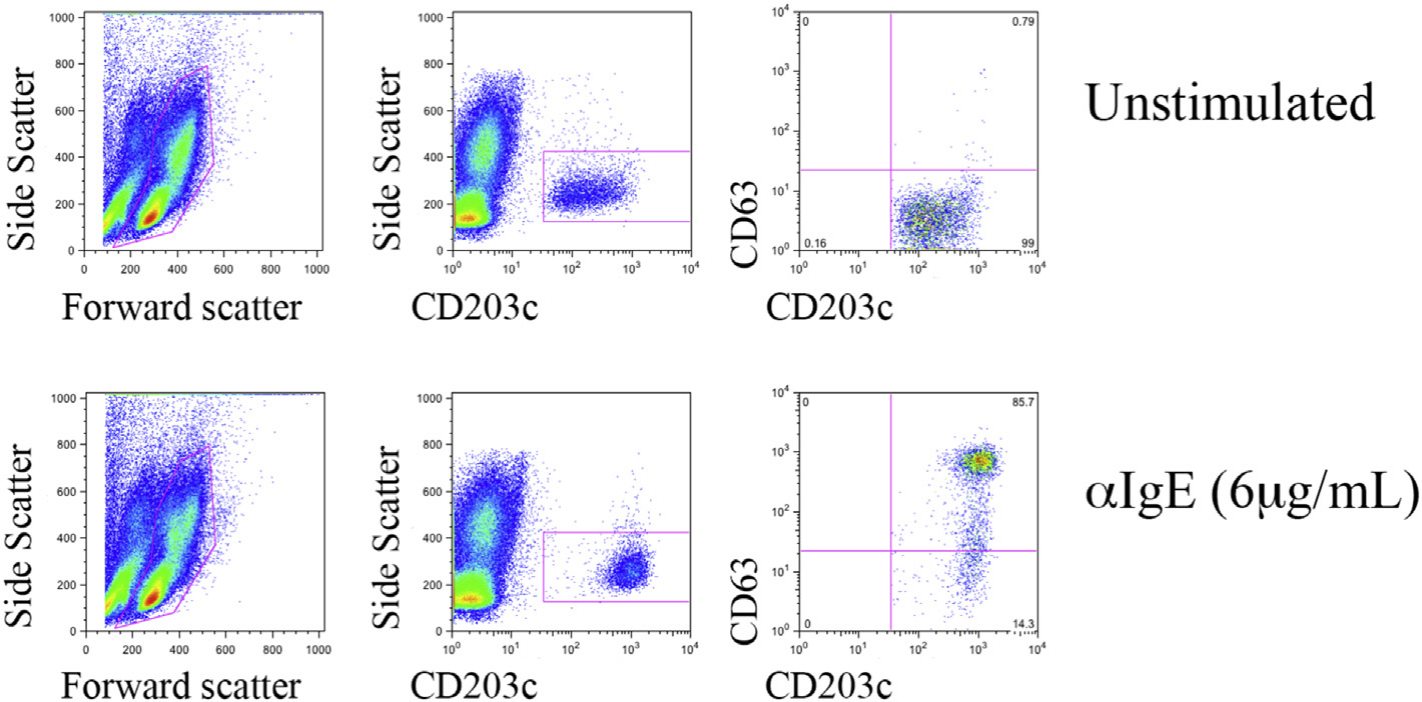
Gating strategy to determine basophil activation by flow cytometry, comparing unstimulated and anti-IgE–stimulated PBMC.

Sera containing IgG anti-IgE specific autoantibodies were tested for their ability to activate blood basophils from a single atopic donor using a standard flow cytometric basophil activation assay. As a positive control, incubation of the donor basophils with polyclonal, exogenous IgG anti-IgE covering the concentration range observed in the sera of the study subjects induced concentration-dependent activation as measured by increased CD63 expression (from 3.15% to 85.6%; Fig 1, *B*; and see Fig E3 in this article's Online Repository at www.jacionline.org). To examine autoantibody activity, these same donor basophils were incubated with subjects' serum samples. Basophil activation was defined as >2.67% CD63^+^ cells, or 3 standard deviations above the mean percentage of basophils expressing CD63 following incubation with the donor's own serum under identical conditions as a baseline. According to these criteria, sera from 2 non-atopic asthmatic subjects (NAA7, NAA18) and 3 atopic asthmatic subjects (AA5, AA8, AA14) were able to activate basophils directly, whereas the others were not (Fig 1, *C*). This activity did not equate with elevated IgG anti-IgE autoantibody concentrations: not all basophil-activating sera contained high concentrations of IgG anti-IgE (Fig 1: AA8), while not all high IgG anti-IgE sera activated basophils (Fig 1: NAA2, NAA16, AA12).

### Modification of allergen-induced basophil activation by IgG anti-IgE autoantibodies

**Fig 2:**
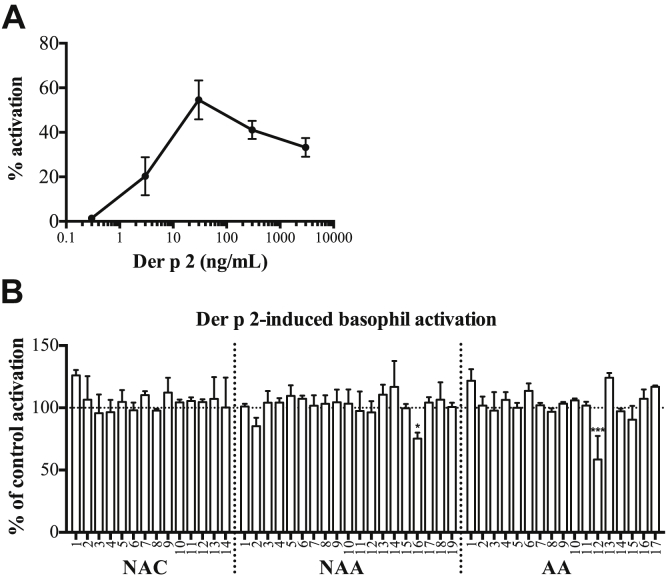
Concentration/response curve of blood basophils from a *Der p 2*–sensitized atopic donor to *Der p 2* allergen *in vitro***(A)**. Response of the same basophils to *Der p 2* 30 ng/mL pre-incubated with all sera from Fig 1 normalized to baseline (pre-incubation of the cells with the donor's own serum) **(B)**. *Bars* represent the mean/SD of at least 3 independent experiments.

**Fig E4:**
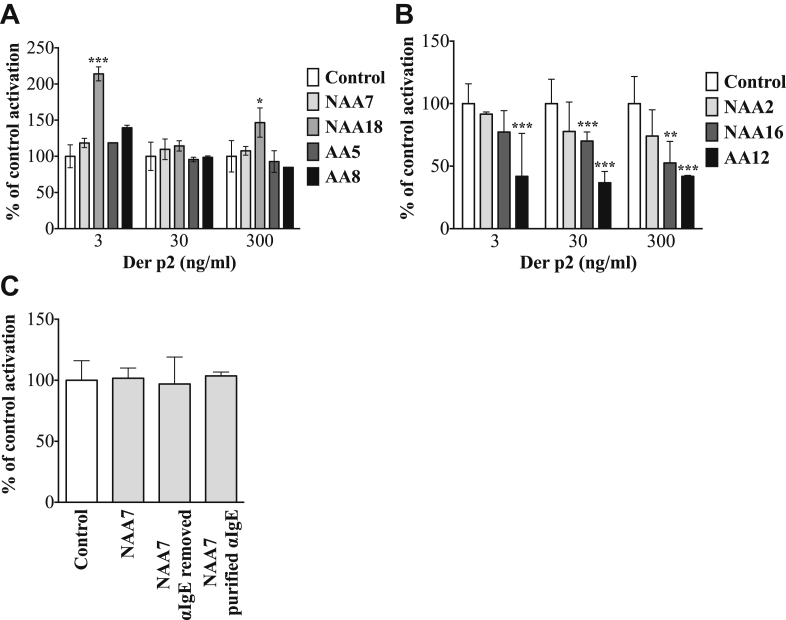
Response of blood basophils stimulated with *Der p 2* (3 to 300 ng/mL), pre-incubated with 4 sera containing IgG anti-IgE autoantibodies and basophil-activating activity **(A)** and 3 sera containing IgG anti-IgE autoantibodies without basophil-activating activity normalized to baseline (pre-incubation of the cells with the donor's own serum) **(B)**. Effects of the non-inhibitory serum NAA7 following the removal and purification of IgE binding proteins on *Der p 2* (30 ng/mL)–induced basophil activation **(C)**. *Bars* represent the mean/SD of 3 independent experiments. ∗*P* < .05, ∗∗*P* < .01, and ∗∗∗*P* < .001.

Considering the above data, we examined the ability of sera from all of the donors to modify allergen-induced activation of blood basophils from the same single atopic donor who was sensitized to the major dust mite allergen *Der p 2*. Following pre-incubation of the donor's basophils with the donor's own serum, *Der p 2* induced concentration-dependent basophil activation (Fig 2, *A*). None of the 5 basophil-activating sera, when pre-incubated with the same donor basophils under identical conditions, significantly reduced the degree of basophil activation induced by a concentration of *Der p 2* optimal for this donor (Fig 2, *B*) and a range of higher and lower concentrations (Fig E4, *A*, in this article's Online Repository at www.jacionline.org: paucity of sample precluded further analysis of serum from subject AA14). In contrast, sera from 2 subjects (NAA16 and AA12), which did not directly increase basophil activation, significantly inhibited allergen-induced activation, while serum from a third subject (NAA2) exhibited an inhibitory effect of borderline significance (Fig 2, *B*, and Fig E4, *B*, in this article's Online Repository at www.jacionline.org).

### Depletion of total IgG and IgE-binding proteins from inhibitory sera abolished inhibition of allergen-induced basophil activation

**Fig 3:**
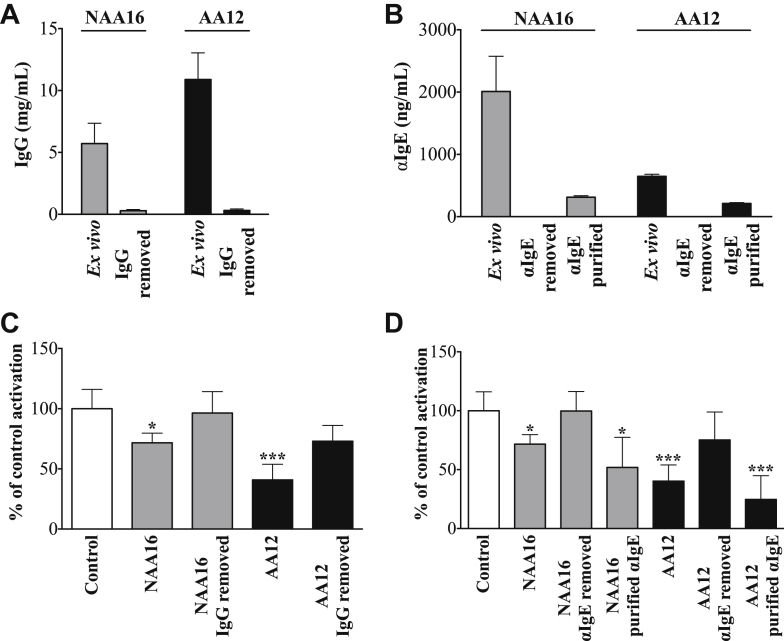
Total IgG concentrations in 2 test sera *ex vivo* and following protein G depletion **(A)**. IgE-binding proteins *ex vivo* and following depletion and isolation of IgE binding proteins **(B)**. Effects of sera *ex vivo* and following IgG depletion **(C)** and removal and purification of IgE binding proteins **(D)** on *Der p 2* (30 ng/mL)–induced basophil activation normalized to baseline (using the donor's own serum). *Bars* represent the mean/SD of 3 independent experiments. ∗*P* < .05 and ∗∗∗*P* < .001.

To further characterize the observed inhibitory activity of the sera from subjects NAA16 and AA12 on allergen-induced basophil activation, we depleted either IgG (Fig 3, *A*) or IgE-binding proteins (Fig 3, *B*). Both maneuvers essentially abolished the ability of the sera to inhibit allergen-induced basophil activation, whereas purified IgE-binding proteins retained this activity in full (Fig 3, *C* and *D*). As a negative control to demonstrate that depletion of IgG or isolation of IgE-binding proteins did not generate basophil modifying activity *per se*, we showed that similar extracts from the non-inhibitory serum NAA7 showed no significant effects on allergen-induced basophil activation (Fig E4, *C*).

### Allergen binding to IgE is inhibited by IgG anti-IgE antibody containing sera

**Fig 4:**
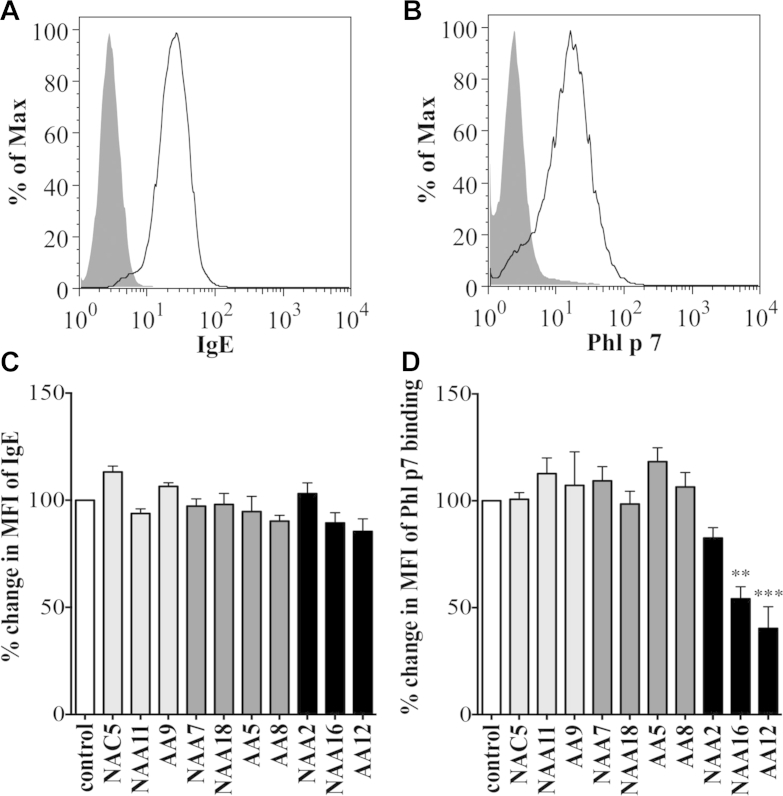
Binding of recombinant *Phl p 7*-specific IgE to FcεRI on RBL-SX38 cells **(A)**. *Phl p 7* binding following further incubation with specific allergen (representative of 3 independent experiments) **(B)**. Changes in surface-bound IgE **(C)** and surface-bound *Phl p 7***(D)** on RBL-SX38 cells pre-incubated with recombinant, anti-*Phl p 7* IgE test sera then *Phl p 7* compared with no serum control. *Bars* represent the mean/SD of 3 independent experiments. ∗∗*P* < .01 and ∗∗∗*P* < .001.

To begin to address the mechanisms of inhibition of allergen-induced basophil activation by the inhibitory sera NAA16 and AA12, we used the rat basophilic cell line RBL-SX38, which expresses surface human FcεRI, and recombinant IgE specific for the timothy grass allergen *Phl p 7*.^20^ Flow cytometric analysis confirmed binding of recombinant IgE anti-*Phl p 7* to the FcεRI expressed on the surface of the RBL-SX38 cells (Fig 4, *A*). Similarly, subsequent addition of fluorochrome-tagged, recombinant *Phl p 7* to the IgE-coated cells resulted in its binding to this specific antibody (Fig 4, *B*). Pre-incubation of IgE-bound RBL-SX38 cells with 4 of the sera that activated basophils directly *ex vivo* but did not modify allergen-induced basophil activation *ex vivo* (NAA7, NAA18, AA5, and AA8; Fig 2, *B*: we were unable to re-test serum AA14 owing to paucity of sample), or 1 serum sample from a donor in each group selected to contain anti-IgE at the threshold of the mean concentration observed in the controls with no basophil activating activity (NAC5, NAA11, AA9) prior to the addition of *Phl p 7* did not significantly alter surface IgE or *Phl p 7* binding (Fig 4, *C* and *D*). In contrast, 2 of the sera that inhibited allergen-induced basophil activation *ex vivo* (NAA16 and AA12, Fig 2, *C*) significantly reduced binding of recombinant allergen but not allergen-specific IgE to the RBL-SX38 cells (Fig 4, *C* and *D*).

## Discussion

We describe a novel and quantitative technique for the measurement of serum IgG anti-IgE autoantibodies. Hitherto these have been identified indirectly using histamine release assays,^23^ or expressed as arbitrary optical density readings in uncalibrated ELISA.^8^, ^24^ Using recombinant IgE as the capture antigen and a pan-anti-human IgG polyclonal antibody for detection, we have been able to calibrate the ELISA using known concentrations of omalizumab, a monoclonal IgG anti-IgE antibody.

Using this assay, we show that there is a subset of asthmatic patients who, regardless of conventional atopic status, have circulating IgG anti-IgE autoantibodies in excess of the range observed in a group of non-asthmatic control subjects, although this is not a consistent finding in the asthmatic subjects and does not permit a clear statistical distinction between the groups. Nevertheless, these observations are of great interest to us from the point of view of a possible role for IgE-mediated mechanisms in asthma, since we have previously reported data suggesting that IgE is expressed to excess in the bronchial mucosa of asthmatic patients regardless of conventional atopic status,^25^, ^26^ and the additional presence of systemic IgG anti-IgE autoantibodies capable of directly activating cells bearing sufficient surface-bound IgE^27^ could represent a mechanism for local, allergen-independent disease exacerbation. Equally significantly, however, our data also show that some IgG anti-IgE autoantibodies actually inhibit allergen-induced activation of basophils expressing surface allergen-specific IgE, at least partly by inhibiting the binding of this IgE to allergen. This may explain why, in this and previous studies, measured concentrations of these antibodies correlate poorly with their clinical effects,^28^, ^29^ and also why they do not necessarily cause disease.^28^ More fundamentally, though, they may at least partly explain phenomena such as why some atopic subjects who manufacture allergen-specific IgE do not develop allergic disease, and why in some patients with asthma, who may manufacture IgE inhibitory IgG endogenously, exogenously administered IgG anti-IgE therapy such as with omalizumab proffers little additional clinical benefit. These questions are readily addressable in future studies.

It is not clear what regulates the production of IgG anti-IgE autoantibody in individual subjects. We observed no correlation between the production of IgG anti-IgE antibodies and the production of other potentially pathogenic IgG autoantibodies such as anti-nuclear autoantibodies (see Fig E2, *B*). Similarly, and in contrast to certain earlier studies,^13^, ^30^ we observed no correlation in individual subjects between serum total IgE and IgG anti-IgE concentrations (see Fig E2, *A*). This may reflect low subject numbers, interference by IgE-specific autoantibodies with the determination of total IgE using different techniques,^31^ or a true lack of any relationship.

Although we studied the properties of IgG anti-IgE autoantibodies from considerable numbers of non-atopic controls and non-atopic and atopic asthmatic subjects in this study, it is clearly arguable that, as in all similar studies, we may not have defined all of the properties of these antibodies or their relative incidence in the entire source populations in an unbiased manner. Having said this, we observed that in no case was their binding inhibited by prior binding of the IgE to its high-affinity receptor FcεRI, suggesting that these IgG species are able to bind to IgE already attached to its high-affinity receptor *in vivo*. This is in contrast to omalizumab, which binds only free IgE. Our findings are congruent with previous studies^9^ suggesting that the majority of IgG anti-IgE autoantibodies recognize the Cε2 or Cε4 domains of IgE, and not the Cε3 domain to which omalizumab is thought to bind^32^ and which is inaccessible when the IgE is bound to its high- or low-affinity receptor. The fact that complete removal either of total IgG or of total IgE binding proteins abolished basophil-modifying activity, whereas the purified IgE binding proteins retained it, is consistent with the hypothesis that it is entirely attributable to IgG anti-IgE. Furthermore, that fact that such activity did not “appear” in fractionated serum from subjects not showing it *ex vivo* (Fig E4, *C*) excludes the possibility that it is an artefact of the separation procedure. Nevertheless, we cannot completely exclude the possibility that some basophil-modifying activity may have reflected the alternative or additional presence of IgG anti-FcεRI autoantibodies.^27^, ^33^, ^34^, ^35^ We have been able to detect such antibodies by further modification of our *in vitro* assays, and further exploration of the properties of these antibodies is the subject of an ongoing further study. The unprecedented potential protective effect of some of these antibodies, however, is of great interest regardless of their precise specificities.

We undertook some preliminary investigations as to possible mechanisms by which inhibitory IgG anti-IgE autoantibodies could reduce allergen-induced basophil activation in our patients. Those we studied did not appear to dissociate IgE from its receptor, a property suggested in previous reports,^36^, ^37^, ^38^ but some did appear to be able to prevent allergen binding to surface-bound specific-IgE, presumably without cross-linking it. This could reflect the inability of IgG anti-IgE in the IgG-IgE-FcεRI complex to interact with another IgE on the cell surface for a variety of reasons.^39^ Further possible mechanisms of basophil inhibition by these antibodies not explored in the present study are also conceivable, such as cross-linking of FcγRIIβ with FcεRI.^40^, ^41^, ^42^ It is worth noting again that the activities of these IgG anti-IgE autoantibodies are quite different from those of omalizumab, which appears to exert its clinical effects simply by preventing binding of IgE to its high- and low-affinity receptors, and do not appear to reduce the intrinsic sensitivity of basophils to activation: on the contrary, recent experiments^43^, ^44^ by MacGlashan and other colleagues suggest that sequestration of IgE during omalizumab therapy renders basophils hypersensitive to activation by cross-linking of reduced surface-bound IgE molecules, at least partly by resetting of FcεRI coupled intracellular signalling.

It is finally apposite to note that production of IgG anti-IgE autoantibodies has been documented in murine “models” of allergen sensitization and tolerance^45^ and in the course of specific allergen immunotherapy in humans,^16^, ^46^ which induces a vigorous IgG_4_ response that could, at least in theory, include IgG anti-IgE autoantibodies. Indeed, 1 of these studies on wasp venom immunotherapy^16^ linked the elevated production of basophil-activating IgG anti-IgE autoantibodies with treatment failure. The experiments in mouse models and immunotherapy in humans have led to speculations about activating but not inhibitory antibodies, which may also be prevalent.

In summary, inhibitory IgG anti-IgE autoantibodies may contribute to a natural regulatory mechanism that could conceivably influence the severity and presence or absence of diseases with IgE-mediated mechanisms, including asthma and allergic rhinitis, and the outcomes of therapeutic processes such as allergen immunotherapy and therapy with exogenous IgG anti-IgE. This demands further, potentially extremely exciting, research.

**Key messages**

• IgE-specific autoantibodies are detectable in atopic and non-atopic asthmatic subjects and controls. They bind to IgE regardless of whether or not it is bound to its high-affinity receptor.
• Some of these autoantibodies activate basophils, whereas others inhibit allergen-induced basophil activation.
